# *In vivo* structural and functional assessment of optic nerve damage in neuromyelitis optica spectrum disorders and multiple sclerosis

**DOI:** 10.1038/s41598-019-46251-3

**Published:** 2019-07-17

**Authors:** Marco Vabanesi, Marco Pisa, Simone Guerrieri, Lucia Moiola, Marta Radaelli, Stefania Medaglini, Vittorio Martinelli, Giancarlo Comi, Letizia Leocani

**Affiliations:** 1 University Vita-Salute San Raffaele, Milan, Italy; 2 Department of Neurology and Institute of Experimental Neurology (INSPE), San Raffaele Hospital Scientific Institute, Milan, Italy

**Keywords:** Multiple sclerosis, Visual system

## Abstract

Early detection of neuromyelitis optica spectrum disorders (NMOSD), especially after optic neuritis, a presenting manifestation commonly observed also in multiple sclerosis (MS), is crucial for timely treatment and prognosis. Integrated visual pathway assessment with optical coherence tomography (OCT) and visual evoked potentials (VEP) may help in this task, showing *in vivo* different pathophysiological backgrounds. We evaluated combined VEP and OCT in a cross-sectional, single-centre study assessing 50 consecutive NMOSD patients, 57 MS patients and 52 healthy controls. After optic neuritis, VEP were more frequently absent in NMOSD compared to MS; most NMOSD eyes with recordable VEP showed prolonged latency, but extreme latency delays were less common than in MS. OCT showed predominantly axonal involvement in NMOSD, with 88% eyes (95% CI: 69–97%) displaying retinal nerve fibre layer thickness <60 µm even after first optic neuritis episode. Accuracy of OCT was further enhanced by combination with VEP into a new Z-score derived OCT-VEP index, measuring prevalence of axonal damage or demyelination. Our results suggest that integrated optic nerve assessment may elucidate differences in optic neuritis pathophysiology; conduction slowing with relatively preserved nerve fibre layer suggests MS, while severe neuroaxonal loss after optic neuritis, often hindering VEP response, characterizes NMOSD.

## Introduction

Recognizing different patterns of damage between neuromyelitis optica spectrum disorders (NMOSD) and multiple sclerosis (MS), especially in patients experiencing optic neuritis (ON), is an important challenge, both for researchers and clinicians, as the two diseases share many clinical and paraclinical features, but differ in critical aspects, first of all prognosis and therapy.

Optical coherence tomography (OCT) studies in NMOSD published since 2008 have shown thinner retinal nerve fibre layer in NMOSD after ON compared to healthy controls^1–4^ and compared to MS eyes with history of ON^1^. In NMOSD eyes without history of ON, however, there are contrasting evidences about OCT involvement, with some studies^5,6^ showing subclinical abnormalities, but not other ones^1,3,7,8^.

Visual evoked potentials (VEP) in NMOSD have been less extensively studied to date^9–11^, with just one published study in Caucasian population^11^. Available results show conflicting evidence about the predominant pattern of VEP involvement in NMOSD patients, whether similar to MS one (with demyelination prevailing on axonal conduction impairment)^11^ or with specific axonal pattern^10^. Still to be elucidated is the relationship between functional and structural measures of optic nerve in NMOSD^12^.

It is known from literature that eyes of MS patients show subclinical RNFL thinning regardless of acute ON events along the course of disease^13,14^. It is not clear whether NMOSD presents with similar subclinical changes.

The aim of the present study was to explore the value of optic nerve structural and functional metrics, alone or in combination, in characterizing NMOSD and differentiating it from MS, in eyes with and without history of optic neuritis.

## Methods

### Protocol approval and patient consents

This was a single-centre, observational, cross-sectional study. Participants were recruited from the Department of Neurology of our Institute. The study, approved by San Raffaele Hospital Scientific Institute Ethical Committee, followed the principles of the Declaration of Helsinki. Written informed consent was obtained from all participants.

### Participants

The participants in the study consisted of 50 consecutive patients with NMOSD, referring to our centre between December 2013 and May 2015, 57 patients with MS randomly selected from our MS centre population, matched for optic neuritis (ON) frequency and disease duration, and 52 healthy controls with comparable age and gender distribution.

All patients had their diagnosis confirmed by their treating neurologists. Diagnosis of MS was based on the 2010 Polman criteria^15^. Diagnosis of NMOSD was based on the 2015 Wingerchuk criteria^16^. Exclusion criteria were the presence of other neurological diseases, concurrent cardiovascular or eye diseases with potential influence on visual function. Eyes with history of ON within last 6 months were excluded from analyses with OCT.

Healthy controls were selected among hospital staff and patients’ families. Inclusion criteria were negative history for neurological, eye and cardiovascular diseases with potential influence on visual function, best corrected high-contrast visual acuity at least 0.8, maximum refraction correction between −6 and +3 spherical dioptres.

### Data collection

For each participant, we collected clinical data (diagnosis, antibody status, Expanded Disability Status Scale [EDSS], previous medical history including ON); visual acuity, pattern-reversal VEP and OCT were taken the same day. NMOSD patients were screened for anti-AQP4 and anti-MOG antibodies. AQP4 antibody testing was performed with indirect immunofluorescence on primate cerebellum and cell-based assay. Two AQP4-negative subjects testing positive for anti-MOG antibodies were excluded from the study.

Visual acuity testing was performed with high-contrast (HCVA) and low-contrast (2.5% and 1.25%) Early Treatment of Diabetic Retinopathy Study (ETDRS) tables, according to ICO recommendations^17^; decimal visual acuity and number of ETDRS table lines read were recorded.

Optical coherence tomography was recorded according to APOSTEL recommendations^18^ using spectral domain OCT Spectralis (Heidelberg Engineering, Heidelberg, Germany; software v5.8), with peripapillary RNFL raster scans, taken without pupil dilation, centred on the optic nerve head (ART 100 frames, diameter 12°). OSCAR-IB criteria were assessed in all RNFL scans with manual review by a single experienced OCT operator^19^.

Monocular pattern-reversal VEP with 15′ and 30′ achromatic checks were recorded according to ISCEV standard^20^ over Oz (10–20 international system), with Cz as reference. Standard stimulus was generated by Micromed Pattern 10 (Micromed, Mogliano Veneto, Italy) device and visualized through LCD monitor. Subjects wear corrective lenses if needed for distance vision. Pattern inversion frequency was 1.7 Hz; at least 30 epochs free from artefacts, contributing to the final average, were recorded; 2–3 consecutive averages were collected for each eye. VEP reproducible components N75, P100 and N145 were marked by the recording technician at the earliest peak of the 2–3 recordings. All measurements were verified by two expert neurologists. P100 latency (when measurable) and inter-peak amplitude were analysed.

Range of normality for VEP and OCT parameters studied was derived from an internal normative, developed on healthy controls, with threshold at 2.5–97.5% percentile. VEP amplitude lower than 3 µV was considered reduced; VEP latency was considered delayed if higher than 120 ms in people under 40 years old, 125 ms in people aged 40–49 years, 130 ms in older people. Peripapillary RNFL thickness lower than 84 µm was classified as reduced. For integrated OCT-VEP analyses, when VEP were absent, amplitude was imputed at 0 µV and latency at 140 ms.

### Statistical analysis

VEP and OCT data were grouped by patient diagnosis and history of optic neuritis: NMOSD ON+, NMOSD ON−, MS ON+, MS ON−. Analyses were performed with R statistical environment (version 3.3, R Foundation for Statistical Computing, Vienna, Austria). Categorical variables and proportions were evaluated with Fisher’s exact test. Quantitative data were assessed with Wilcoxon rank-sum test or its parametric equivalents according to data distribution. Regression analysis was performed with generalized mixed linear models (using *lme4* packages) and generalized estimating equations (GEE) models, accounting for within-subject effects (using *geepack, multcomp* and *BSagri* packages); subsequent pairwise comparisons were performed with Wald test. For all tests the alpha level was set at 0.05.

## Results

Demographic, clinical and baseline data of study groups are shown in Table 1.

**Table 1:** Demographic and clinical data.

Subjects	NMOSD (*N* = 50)	vs. (*p-value*)^(a)^	MS (*N* = 57)	HC (*N* = 52)
Gender, F/M (female %)	43/7 (86.0%)	0.007	38/19 (66.7%)	37/15 (71.1%)
Age, yr (mean, SD)	44.9 ± 12.7	0.11	38.0 ± 10.0	37.5 ± 17.0
Disease duration, yr (mean, SD)	6.6 ± 6.8	0.71	7.1 ± 7.2	—
EDSS (median, range)	3.5 (1.5–8.5)	<0.001	1.5 (1.0–6.0)	—
% Ab anti-AQP4 positive % Ab anti-MOG positive	29/45 (64.4%)^(b)^ 0/45 (0.0%)^(b,c)^	—	—	—
Nr. eyes studied with VEP	98^(d)^		114	104
Nr. eyes studied with OCT — of which included (ON ≥6 months)	72 59		114 104	104 104
Nr. eyes with history of ON (%) — of which single ON episode Nr. ON episodes per eye (mean, SD)	56/100 (56.0%) 35/56 (62.5%) 0.91 ± 1.11	0.26 0.02 0.07	55/114 (48.2%) 45/55 (81.8%) 0.59 ± 0.70	—

NMOSD: neuromyelitis optica spectrum disorders. MS: multiple sclerosis. HC: healthy controls. EDSS: Expanded Disability Status Scale. AQP4-Ab: antibodies anti–aquaporin-4. VEP: visual evoked potentials. OCT: optical coherence tomography. ON: optic neuritis. — ^(a)^Wilcoxon rank-sum test was used for numerical variables and chi-square test for proportions. ^(b)^Antibody testing was not available for five patients with NMOSD. ^(c)^Two MOG-positive patients were not included in the study. ^(d)^Two VEP recordings in subjects with NMOSD were not included due to poor compliance.

Table 2 shows main results about visual acuity testing, VEP and OCT parameters in study groups. Antibody testing was available for 45/50 NMOSD subjects. NMOSD subjects did not show differences in OCT and VEP parameters on the basis of anti-AQP4 antibody serostatus (GEE model, p = 0.87 for VEP latency, p = 0.33 for OCT RNFL).

**Table 2:** Structural and functional optic nerve parameters.

Eyes	NMOSD ON+ (*N* = 56)	NMOSD ON− (*N* = 44)	MS ON+ (*N* = 55)	MS ON− (*N* = 59)
Decimal HCVA (mean, SD)	0.35 ± 0.35	1.00 ± 0.17	0.91 ± 0.33	0.98 ± 0.16
% low vision (HCVA < 0.32)	30/56 (53.6%)	0/44 (0.0%)	4/55 (7.3%)	0/59 (0.0%)
HCVA (nr. ETDRS rows read)	3.8 ± 4.5	11.1 ± 0.9	10.0 ± 2.6	10.9 ± 0.7
2.5% contrast VA (nr. rows)	0.6 ± 1.6	4.8 ± 2.1	3.0 ± 2.9	4.1 ± 2.4
1.25% contrast VA (nr. rows)	0.3 ± 1.3	3.7 ± 2.7	1.5 ± 1.9	2.0 ± 1.8
**Visual evoked potentials**				
Nr. eyes included	54^(a)^	44	55	59
15′ VEP: % absent	34/54 (63.0%)	1/44 (2.2%)	4/55 (7.3%)	1/59 (1.7%)
15′ VEP: mean latency (ms)	137.6 ± 13.2	122.8 ± 7.4	134.1 ± 18.1	125.2 ± 13.3
15′ VEP: % increased latency — of which >150 ms	18/20 (90.0%) 1/20 (5.0%)	11/43 (25.6%) 0/43 (0.0%)	33/51 (64.7%) 9/51 (17.6%)	27/58 (46.6%) 4/58 (6.9%)
15′ VEP: mean amplitude (µV)	5.0 ± 3.0	8.1 ± 4.5	6.5 ± 4.3	7.5 ± 5.0
15′ VEP: % reduced amplitude	5/20 (25.0%)	5/43 (11.6%)	9/51 (17.6%)	9/58 (15.5%)
30′ VEP: % absent	30/54 (55.5%)	0/44 (0.0%)	3/55 (5.5%)	0/59 (0.0%)
30′ VEP: mean latency (ms)	135.3 ± 15.2	120.1 ± 7.6	130.3 ± 16.3	121.6 ± 13.7
30′ VEP: % increased latency	20/24 (83.3%)	9/44 (20.4%)	33/52 (63.4%)	19/59 (32.2%)
**Optical coherence tomography**				
Nr. eyes included	25	34	45	59
OCT: mean RNFL (µm)	44.1 ± 12.9	94.4 ± 11.9	78.7 ± 13.4	88.1 ± 12.0
OCT: % reduced RNFL — of which <60 µm	26/26 (100.0%) 23/26 (88.5%)	6/34 (17.6%) 0/34 (0.0%)	29/45 (64.4%) 3/45 (6.7%)	16/59 (27.1%) 2/59 (3.3%)

ON+: eyes with history of optic neuritis. ON−: eyes without history of optic neuritis. VA: visual acuity. HCVA: high-contrast visual acuity. ETDRS: Early Treatment for Diabetic Retinopathy Study. RNFL: retinal nerve fibre layer. — ^(a)^Two VEP recordings in subjects with NMOSD were not included due to poor compliance.

### VEP and OCT in eyes with ON history

Among eyes with ON history, the frequency of absent 15′ VEP was significantly higher in NMOSD (63.0% [95% CI: 49.6–74.6%]) compared to MS (7.3% [CI: 2.9–17.3%], p < 0.001, Fisher’s exact test).

**Figure 1:**
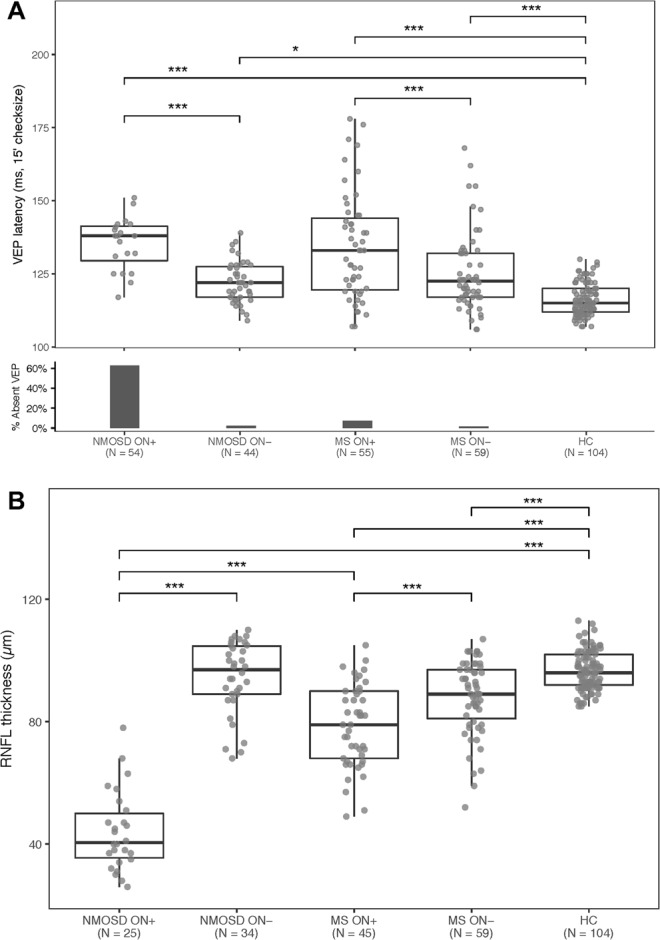
Distribution of visual evoked potentials (VEP) latency (panel A) and retinal nerve fibre layer (RNFL) thickness (panel B). Generalized estimating equation (GEE) model (p < 0.001 overall). Asterisks reflect significance of pairwise comparisons after GEE (*p < 0.05, **p < 0.01, ***p < 0.001). In Panel A, only eyes with non-absent VEP are shown. HC: healthy controls.

As shown in Fig. 1A, average P100 latency delay was observed in ON+ eyes of both NMOSD and MS patients compared to ON− eyes and to healthy controls (GEE); average latency did not differ between NMOSD and MS (p = 0.97). In ON+ eyes, VEP were delayed in 18/20 (90.0%) NMOSD eyes with recordable VEP (18/54, 33.3% of all eyes) and in 64.7% (33/51) MS eyes with recordable VEP (33/55, 60.0% of all eyes). A smaller proportion of severe P100 latency delay (over 150 ms) was observed in NMOSD compared to MS ON+ eyes (p = 0.016, Fisher). The analysis of 30′ check-size VEP produced analogous results (not shown).

Peripapillary RNFL thickness measured with OCT was decreased in both NMOSD and MS ON+ eyes, compared to ON− eyes and to healthy controls (Fig. 1B). Compared to ON− eyes, average RNFL thinning was 50.3 ± 3.9 µm in NMOSD and 9.4 ± 2.1 µm in MS; mean RNFL thickness was lower in NMOSD than in MS (44.1 vs. 78.7 µm, p < 0.001, GEE).

At receiver operating characteristic (ROC) analysis, a threshold of 60 µm was determined to have optimal accuracy to separate NMOSD from MS ON+ eyes, with values lower than 60 µm having 88% (95% CI: 69–97) sensitivity and 93% (CI: 81–98) specificity for NMOSD diagnosis. The same cut-off was found also when limiting the analysis to eyes with a single previous ON episode (*N* = 14 for NMOSD, *N* = 36 for MS), with sensitivity of 93% (95% CI: 64–100) and specificity of 92% (CI: 76–98). No significant effect of number of ON episodes on RNFL was observed in subgroup analysis.

**Figure 2:**
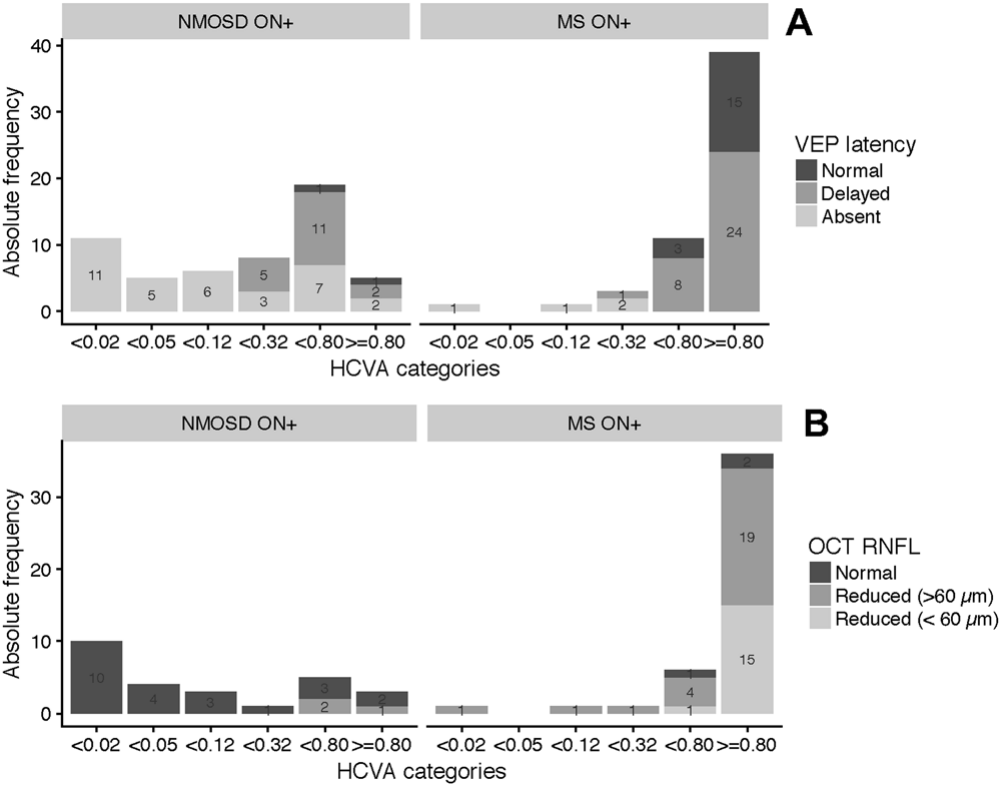
Relationship of VEP latency (Panel A) and RNFL thickness (Panel B) with visual acuity. Decimal high-contrast visual acuity (HCVA) classes: (near)-blindness (<0.02); profound (<0.05), severe (<0.12), moderate (<0.32), mild (<0.80) visual impairment, normal vision (≥0.80). Stratified by VEP latency and OCT RNFL thickness category.

Low vision, defined by decimal high-contrast visual acuity (HCVA) less than 0.32^17^, was significantly more common in NMOSD eyes (p < 0.001, Fisher’s exact test) than in MS. When analysing VEP and RNFL categories in ON+ eyes, stratified by range of visual performance^17^ (Fig. 2), worse visual performance was accompanied by an increased frequency of absent VEP and reduced RNFL thickness in both NMOSD and MS eyes. However, in NMOSD, even for comparatively good visual performance, very severe RNFL thinning (<60 µm) was observed, while MS eyes with severe visual impairment did not show comparable grades of RNFL reduction. Statistical analysis confirmed the significant effect of NMOSD diagnosis (p < 0.001, GEE) in changing the relationship between RNFL thickness and HCVA after ON.

We compared the contribution of RNFL thickness and HCVA to diagnosis of NMOSD after ON with a multivariate logistic model, which showed RNFL was the only significant predictor (p < 0.001), encompassing the effect of visual acuity: NMOSD diagnosis becomes more probable, with OR 1.16 (95% CI: 1.08–1.29), for every 1 µm RNFL reduction.

Relationship between VEP latency increase and HCVA was affected too by NMOSD diagnosis, with trend for significance considering only the 20 NMOSD ON+ eyes with recordable VEP (p = 0.12, GEE) and much stronger statistical evidence (p < 0.001) including also ON− eyes.

### VEP and OCT in eyes without ON history

Mean VEP latency observed in MS eyes without ON history was increased compared to healthy controls (p < 0.001, GEE); RNFL thickness was reduced vs. controls (p < 0.001).

In NMOSD ON− eyes, RNFL thickness did not differ from controls (p = 0.47), but VEP latency displayed an average P100 delay of 6.6 ± 1.4 ms compared to controls (p < 0.001, GEE); comparison of latency distributions (median [IQR]: 122 [10.8] ms in NMOSD vs. 115 [7.0] ms in controls) confirmed that this finding was not due to effect of outliers (not shown).

In our MS cohort we observed a correlation with disease duration, with 0.68 ± 0.30 ms P100 delay (p = 0.022, GEE) and 0.87 ± 0.21 µm RNFL thinning (p < 0.001) for every year of disease duration, while controlling for age and fellow eye ON status. In NMOSD we found VEP latency increasing 0.24 ± 0.07 ms for every year of age (p < 0.001, GEE), while relationship with disease duration, controlling for patient age, showed a trend for significance (+0.28 ± 0.17 ms per year, p = 0.10); no significant role for fellow eye ON status was found (p = 0.29). No statistical relationship between RNFL thickness and age or disease duration in our NMOSD cohort was found.

### Integrated VEP-OCT measures

**Figure 3:**
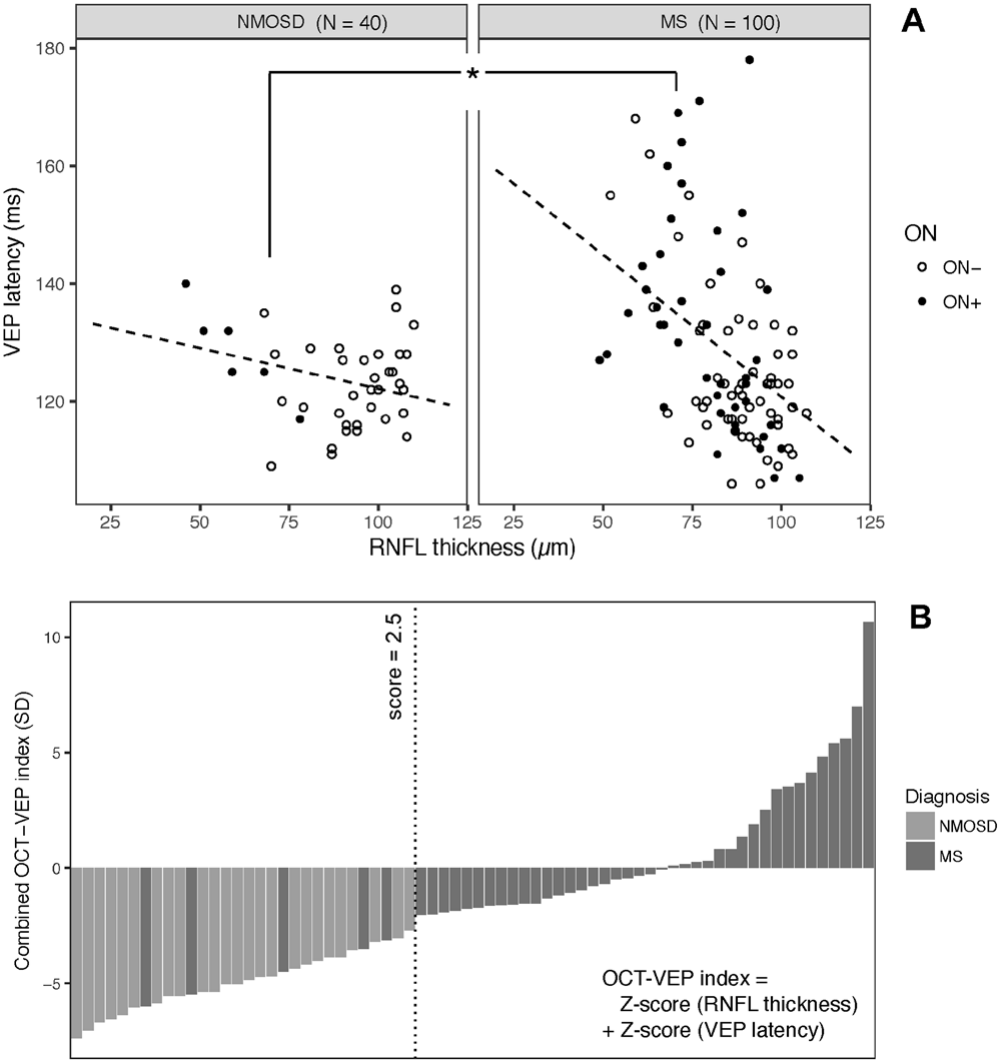
Combined OCT-VEP analysis. In *Panel A*, relationship between RNFL thickness and VEP latency is assessed with a GEE model. Only eyes with non-absent VEP, at least 6 months since last optic neuritis (ON), are included. The linear relationship is significantly different in NMOSD vs. MS (Wald test, p = 0.02). In *Panel B*, only eyes with history of optic neuritis are included; every bar represents a single eye. OCT-VEP index is calculated as algebraic sum of Z-score for RNFL thickness and Z-score for VEP latency; threshold of −2.5 (dotted line) has the highest accuracy to discriminate NMOSD from MS.

A GEE regression model was designed to compare in NMOSD and MS eyes, including both ON+ and ON− eyes, the relationship between VEP latency and RNFL thickness; for 1 µm RNFL reduction, VEP latency was delayed on average by 0.48 ± 0.10 ms in MS and 0.14 ± 0.08 ms in NMOSD, showing a significant prevalence of axonal involvement in NMOSD vs. MS (p = 0.02, Wald test; Fig. 3A).

A combined OCT-VEP index was calculated as the algebraic sum of VEP latency and RNFL thickness Z-scores of deviation from healthy controls: in our control cohort, average (± SD) VEP latency was 116.2 ± 5.3 ms, while average RNFL thickness was 96.7 ± 6.3 µm. VEP latency delay increases the index, while RNFL reduction reduces it.

Considering only eyes with history of ON, NMOSD patients showed lower OCT-VEP index values compared to MS (−4.99 ± 1.27 in NMOSD vs. 0.64 ± 3.73 in MS; p < 0.001, t-test), also considering only eyes with recordable VEP (−3.86 ± 0.73 in NMOSD vs. 0.15 ± 3.35 in MS; p = 0.006, t-test). A threshold value of −2.5 for combined OCT-VEP index, derived from ROC curve (not shown), had the highest accuracy (100.0% [95% CI: 83.4–100.0] sensitivity and 88.9% [CI: 75.1–95.8] specificity) in differentiating NMOSD from MS after optic neuritis (Fig. 3B).

## Discussion

An important question yet to be fully answered is how to differentiate MS and NMOSD, particularly after optic neuritis, which can be the presenting symptom of both. Multiple clinical features have been described to help formulating a diagnostic hypothesis^21^ and AQP4 antibody testing is the mainstay of differential diagnosis, with high sensitivity and excellent specificity. To complement antibody testing, evoked potentials and optical coherence tomography, widely available with relatively low cost, if validated, may help raising suspicion of NMOSD, particularly in AQP4-antibody negative subjects, and elucidate details on prevailing pathophysiology mechanisms.

### VEP and OCT in eyes with history of ON

In the present study, the frequency of absent VEP in NMOSD after ON, significantly higher than in MS, is consistent with rates between 47% and 64% reported in literature^9,10^. VEP delay in MS ON+ eyes, often persisting after clinical recovery from optic neuritis, has been recognized by decades. Data on VEP latency in NMOSD are less established. In a Japanese cohort studied by Watanabe^9^, P100 was delayed over 121 ms (30′ check-size) in only 1/6 (17%) AQP4+ patients with non-absent VEP response, compared to 28/64 (44%) MS patients with delayed latency. Neto^10^ in Brazilian subjects reported latency delay over a threshold of 117.6 ms (43′ check-size) in 2/20 (10%) NMOSD eyes with recordable VEP; a specific VEP “NMO pattern” with normal latency and isolated amplitude reduction was therefore suggested.

In contrast with these results, in the only study on European population published to date, by Ringelstein and colleagues^11^, considering both ON+ and ON− eyes, 36/74 (49%) NMO eyes with non-absent VEP had P100 latencies equal or higher than 120 ms (41′ check-size). Among eyes with ON history, a higher rate of VEP delay was found, with average latency of 131.2 ± 20.7 ms.

A thorough comparison of VEP latency delays and frequency of absent responses across studies is not possible due to different criteria for patient selection and to technical differences that may influence these measures. Consistently with previous findings^11^, we observed VEP latency delay in almost all NMOSD ON+ eyes with recordable VEP. However, after ON we found a lower frequency of severe latency delay (over 150 ms) in NMOSD than in MS.

In the present study, optic neuritis in NMOSD eyes was associated to an average RNFL loss of 35 µm more than in MS, a strong difference already reported in the literature with values of 24 µm according to Ratchford *et al*.^1^. Moreover, RNFL thickness lower than 60 µm in eyes with ON history was very highly suggestive of NMOSD diagnosis instead of MS, with 69–97% sensitivity and 81–98% specificity. No effect of number of ON events was found, and the result was corroborated by confirmation in the subgroup of eyes with single ON episode history.

Another finding of the present study is the higher performance of RNFL compared to visual acuity in detecting NMOSD patterns. It appears that the relationship between structural integrity, measured with OCT and VEP, and visual outcome (HCVA) is not conserved across different diseases: severe, destructive, axonal damage^22^, unparalleled by demyelination, is suggested in NMOSD from extreme RNFL loss, as well as frequent VEP absence. On the other hand, visual outcome in MS may be linked to severe demyelination with relatively preserved axonal integrity.

### VEP and OCT in eyes without history of ON

This study confirms the finding of VEP latency and RNFL thickness abnormalities in MS ON− eyes already documented in literature^23–25^. With regard to NMOSD, Ringelstein^11^ reported delayed average VEP latency, compared to healthy controls, in NMOSD ON− eyes. Several studies have shown normal RNFL thickness in eyes without history of ON^1,3,7,8^, suggesting that subclinical damage in NMOSD is uncommon. Other studies reported instead axonal damage on optic nerve and retina^5,6^.

While our study did not show any significant RNFL thickness reduction, we reported average 6.6 ms VEP latency increase in NMOSD ON− eyes, not due to outliers effect; the main explanatory hypotheses for this finding could be minor subclinical ON events or chronic microstructural damage impacting on conduction speed. In the latter case, an increasing latency trend with disease duration may be observed; we found non-conclusive data suggesting this trend (p = 0.10), which may be further analysed with longitudinal studies.

### Integrated structural and functional measures

Combination of structural and functional visual pathway assessment techniques showed different prevailing damage patterns in NMOSD vs. MS. In MS, more severe VEP delays were observed facing relatively preserved RNFL, suggesting predominance of demyelination over axonal loss. In NMOSD, severe axonal atrophy predominated, often hindering VEP identifiable responses.

This finding is consistent with the evidence from pathology of direct damage to axons in NMOSD, mediated by anti-AQP4 antibody astrocytopathy^22,26^, while in MS functional transmission impairment and axonal degeneration occurs mainly as a secondary effect of demyelination^27^.

Our results appear overall consistent with the reported observation of a more severe GCIPL (ganglion cell-inner plexiform layer) thinning at macular OCT and milder multifocal VEP latency delay after optic neuritis in 19 NMOSD subjects, compared with MS^28^. Our study further clarifies that more severe axonal atrophy occurs in NMOSD also for equal levels of visual acuity impairment and VEP delay, highlighting different structure-function relationships in the two diseases. With this respect, we quantified the balance between axonal damage and demyelination using a combined OCT-VEP index as a newly proposed marker, which showed promising accuracy in our sample, prompting further validation studies in larger cohorts.

AQP4 antibody status did not have a significant effect on OCT-VEP parameters in our NMOSD cohort, whatever the ON involvement, to suggest the hypothesis that the same pathological process may underlie both AQP4+ and AQP4− NMOSD subtypes.

### Strengths and limitations of the study

There are several potential limitations to our study. First, this was a single-centre study with unblinded analysis and a relatively small number of subjects included, with some heterogeneity in disease severity and duration. Studies in newly diagnosed patients could provide more robust evidence for differences in early disease course. Another limitation comes from the lack of availability of OCT recordings in a subset of NMOSD patients, precluding integrated analyses on those subjects. Severe reduction of visual acuity, particularly represented in the NMOSD group, could reduce the quality of VEP and OCT recordings. The potential advantage of technological advancements in OCT (e.g. faster high-resolution acquisition) and VEP (e.g. multifocal stimulation) over standard methods should be explored.

Despite these limitations, this study correlates *in vivo* structural and functional measures of visual pathway, to inspect the contribution of axonal degeneration and demyelination to MS and NMOSD pathology. A combined structural-functional marker, such as the OCT-VEP index, demonstrated promising accuracy for discrimination of NMOSD from MS after one or more episodes of optic neuritis. Future studies are needed aimed at providing neurologists and neuro-ophthalmologists with extended tools for differential diagnosis and prognosis of optic neuritis.
